# Supplementary material for: The causal role of circulating inflammatory markers in osteoporosis: a bidirectional Mendelian randomized study
Source: Front Immunol. 2024 Jul 18;15:1412298. doi: 10.3389/fimmu.2024.1412298 (PMC11291241; doi:10.3389/fimmu.2024.1412298)
Supplement: Supplementary file 1 [file DataSheet_1.zip › supplementary material/CIM-OSTEOPOROSIS /GCST90274847.csv_forest.pdf]

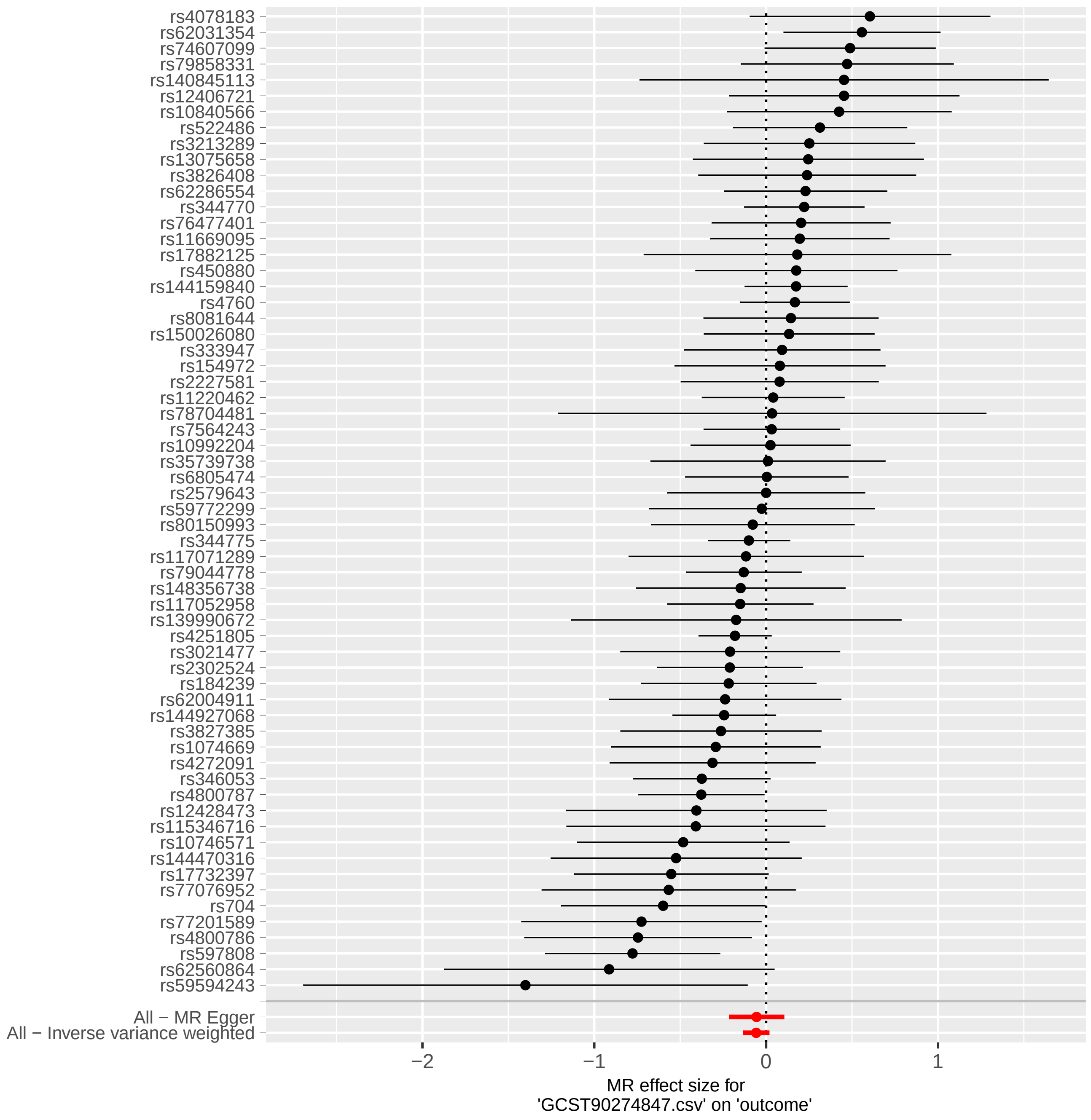
rs4078183
rs62031354
rs74607099
rs79858331
rs140845113
rs12406721
rs10840566
rs522486
rs3213289
rs13075658
rs3826408
rs62286554
rs344770
rs76477401
rs11669095
rs17882125
rs450880
rs144159840
rs4760
rs8081644
rs150026080
rs333947
rs154972
rs2227581
rs11220462
rs78704481
rs7564243
rs10992204
rs35739738
rs6805474
rs2579643
rs59772299
rs80150993
rs344775
rs117071289
rs79044778
rs148356738
rs117052958
rs139990672
rs4251805
rs3021477
rs2302524
rs184239
rs62004911
rs144927068
rs3827385
rs1074669
rs4272091
rs346053
rs4800787
rs12428473
rs115346716
rs10746571
rs144470316
rs17732397
rs77076952
rs704
rs77201589
rs4800786
rs597808
rs62560864
rs59594243
All − MR Egger
All − Inverse variance weighted
−2
−1
0
1
MR effect size for
'GCST90274847.csv' on 'outcome'
